# Individuals with severely impaired vision can learn useful orientation and mobility skills in virtual streets and can use them to improve real street safety

**DOI:** 10.1371/journal.pone.0176534

**Published:** 2017-04-26

**Authors:** Ellen Lambert Bowman, Lei Liu

**Affiliations:** School of Optometry, University of Alabama at Birmingham, Birmingham, Alabama, United States of America; University College London, UNITED KINGDOM

## Abstract

Virtual reality has great potential in training road safety skills to individuals with low vision but the feasibility of such training has not been demonstrated. We tested the hypotheses that low vision individuals could learn useful skills in virtual streets and could apply them to improve real street safety. Twelve participants, whose vision was too poor to use the pedestrian signals were taught by a certified orientation and mobility specialist to determine the safest time to cross the street using the visual and auditory signals made by the start of previously stopped cars at a traffic-light controlled street intersection. Four participants were trained in real streets and eight in virtual streets presented on 3 projection screens. The crossing timing of all participants was evaluated in real streets before and after training. The participants were instructed to say “GO” at the time when they felt the safest to cross the street. A safety score was derived to quantify the GO calls based on its occurrence in the pedestrian phase (when the pedestrian sign did not show DON’T WALK). Before training, > 50% of the GO calls from all participants fell in the DON’T WALK phase of the traffic cycle and thus were totally unsafe. 20% of the GO calls fell in the latter half of the pedestrian phase. These calls were unsafe because one initiated crossing this late might not have sufficient time to walk across the street. After training, 90% of the GO calls fell in the early half of the pedestrian phase. These calls were safer because one initiated crossing in the pedestrian phase and had at least half of the pedestrian phase for walking across. Similar safety changes occurred in both virtual street and real street trained participants. An ANOVA showed a significant increase of the safety scores after training and there was no difference in this safety improvement between the virtual street and real street trained participants. This study demonstrated that virtual reality-based orientation and mobility training could be as efficient as real street training in improving street safety in individuals with severely impaired vision.

## Introduction

The National Eye Institute of the United States of America estimated that 2 million Americans suffered from low vision (visual acuity between 20/40 and 20/200) and 1 million suffered blindness (acuity ≤ 20/200) in 2010 and the numbers will double in 2030 [1, 2]. Low vision has profound negative impacts on the individual’s physical and psychological wellbeing, personal independence, employment and quality of life [3]. On the other hand, only 15% of the individuals with low vision have no usable vision (light perception or worse). The rest have various degrees of usable vision [4]. Rehabilitation is the primary treatment option for individuals with low vision [5]. It provides a wide range of professional services that enable individuals to maximize the use of their remaining vision, to supplement impaired vision with other sensory inputs and to learn alternative strategies to perform daily tasks.

One of the most sought after, high-value low vision rehabilitation items is Orientation and Mobility (O&M) rehabilitation, which teaches special skills to individuals with low vision so that they can travel independently, safely, and swiftly through their environment [6, 7]. These skills are taught in a one-on-one manner between a certified O&M specialist (COMS) and a visually impaired student on real streets or real buildings. While this mode of training has been the standard of care since the end of WWII, its limitations in accessibility and affordability have become more noticeable when a growing portion of the otherwise healthy population suffer from visual impairment. This is because a COMS has to accompany a low vision trainee on the street to ensure her safety throughout training, but the COMS resource is scarce and costly. In 2016, there were only 2666 current O&M certificate holders in the US listed in the Academy for Certification of Vision Rehabilitation and Education Professionals website. Most of them concentrate in Veteran’s Administration blind rehabilitation centers and academic university medication centers. Only 20% of non-Veteran’s Administration low vision rehabilitation entities have at least one COMS working part-time or full-time [5]. Most of the O&M rehabilitation cost comes from COMS’ time. To gain the skills to navigate unfamiliar environment safety, a couple of hundred hours of instruction is needed [6]. The hourly COMS cost varies from $50 to $120 in the US, which is usually not reimbursable by Medicare, Medicaid, or private insurance. Access to COMS and financial burden are main barriers to realizing a low vision individual’s full potential for independent travel.

A large portion of the O&M curriculum is focused on learning to gather information about the environment using the remaining vision and other sensory inputs and to make timely and safe decisions. These sensory, perceptual and cognitive skills are quite different from those used by the individual before his vision is impaired, and thus require instructions and practice. Virtual Reality (VR), a computer generated sensory environment, has great potential to improve current O&M rehabilitation. VR has found successful applications in psychotherapy, orthopedic rehabilitation and neuropsychological rehabilitation [8–14]. Another important area of VR applications is skill acquisition. These typically involve learning high risk skills in risk-free virtual environments, such as learning to fly a plane or to drive a truck in a VR simulator. Surgery simulators have become an integral component in surgical skill training [15–17]. VR has been successfully applied to teaching road safety to children [18, 19] and stroke patients [20, 21]. There has been a sizeable amount of literature on teaching O&M skills to blind individuals who possess no usable vision and have to rely on alternative sensory inputs. It has been shown that these individuals could successfully explore a new virtual acoustic or haptic + acoustic space [22, 23]. Seki and Sato used blind-folded normal participants to demonstrate that training in a virtual acoustic environment could outperform training in a real environment in some O&M skill metrics [24].

In comparison, there is little research on teaching visual skills to individuals with low vision in VR. Kalia, Legge and Giudice asked low vision participants to explore an indoor hallway in four presentation modes, a photo-realistic virtual hallway, a sparse virtual hallway containing only geometric cues of the hallway, a map and the real hallway [25]. The participants’ knowledge of the hallway was tested by map drawing and by walking to specified acoustic targets in the real hallway. Low vision participants showed better knowledge of the hallway from learning the photographic virtual hallway than from learning the sparse virtual hallway. Learning from both virtual hallways were less efficient than learning from a map or the real hallway. This study demonstrated that individuals with low vision could benefit from high quality visual information.

The key issue in VR-based O&M skill training is whether low vision students can use VR-acquired skills to solve mobility problems in real streets. While VR provides a controllable, quantifiable, less stressful and safe environment for learning and practicing potentially dangerous road safety skills, virtual reality is not the same as physical reality. Due to technological and cost restraints, not all aspects of real world can be faithfully simulated in VR. From this point of view, VR is a compromised representation of physical reality. It is of paramount importance to determine if the students can learn anything useful from this compromised representation and, more importantly, to what degree the students can apply the VR-acquired skills to solve problems in the real world. Therefore, an important first step of any VR application development is to determine the efficacy and extent of VR to real world skill transformation. Developers and users of surgical simulators view VR to Operating Room as the gold standard for judging the impact of the simulators on surgical skill training [26]. The same applies to VR training of O&M skills. Many important sensory inputs from the real environment that can potentially influence O&M, such as stereoscopic depth, spatialized sound, vibration on the ground, heat of a car or change of air flow, are technically too complicated or too costly to simulate in a clinically deployable VR simulator. Training in a VR, one does not have to fear the consequence of making a mistake or be embarrassed by not being able to behave like a normally sighted pedestrian. Can low vision students, whose vision is already severely compromised, learn useful O&M skills from such a compromised presentation of the real streets? If they can, will they be able to transfer the skills to the real streets? This study was designed to assess VR skill transferability in individuals with severely impaired vision. We hypothesized that these individuals can learn useful O&M skills from an affordable VR simulator and can efficiently transfer VR-acquired skills to solving real world O&M problems.

## Materials and methods

### Research design

A two-group pre- and post-training design was used to quantitatively assess the transferability of VR-acquired O&M skills. In this design, low vision participants were pseudo-randomly assigned to a virtual street training group and a real street training group, which received O&M skill training in virtual streets presented by a VR simulator and in comparable real streets, respectively. Road safety of all participants were evaluated in real streets before and after training. The primary outcome measure of the research was the change of road safety in real streets before and after training.

### O&M task and skills

The task to be studied was to make a safe decision to cross a signal controlled street. It was chosen because it involved using visual and auditory skills to collect real-time information in a complex and highly dynamic environment and to make a correct decision in a timely manner. A wrong decision can potentially put the traveler in a dangerous situation.

Traffic engineers design pedestrian signal schema such that if a pedestrian leaves the curb during a prescribed period of time after the onset of the WALK sign, he is assured of sufficient time to complete his crossing. Specifically, the pedestrian signal contains two phases. The DON’T WALK phase usually coincides with the red light on the same direction for cars. Any attempt to cross the street in this phase is unsafe. The pedestrian phase has two intervals. The WALK interval, during which the WALK sign or the white walking figure is shown. The WALK interval is typically 7 sec long. It is the time when a slow-average walker must leave the curb in order to have sufficient time to walk to the far side. The pedestrian clearance interval, or FLASHING DON’T WALK interval, during which the pedestrian signal is flashing or counting down, indicates the time needed for a person walking at a speed of 3.5 feet/second or faster to walk across the street [27]. The pedestrian signal provides salient visual information to guide crossing decision making for people who can see it.

**Fig 1:**
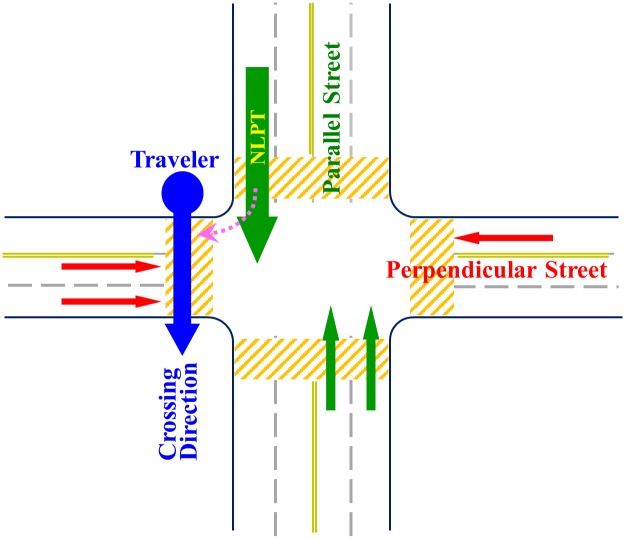
Layout of a cross street intersection and traffics. The traveler is standing at the street corner indicated by the blue circle and intends to cross the perpendicular street in the direction indicated by the blue arrow. Red and green arrows are perpendicular and parallel traffic, respectively. The near lane parallel traffic (thick green arrow, NLPT) comes from behind, over his left shoulder. Right-turn cars (magenta dashed arrow) may share the near lane.

For people who cannot see the pedestrian signal in a street intersection shown in Fig 1, safe crossing is still possible but a different set of skills has to be learned to infer the traffic cycle timing. The commonly taught O&M skill is called the near lane parallel traffic surge (NLPTS). In O&M terminology, a perpendicular street is the street to be crossed (perpendicular to one’s direction of travel). A parallel street is the street that is parallel to one’s direction of travel. Parallel traffic is the traffic that runs on the parallel streets. For each crossing scenario, there is one lane of the parallel traffic that is the closest to the traveler. This is the near lane parallel traffic. Its nearness makes its cars more visible and audible than those in other lanes of parallel traffic. In the crossing scenario shown in Fig 1, the near lane parallel traffic (thick green arrow) comes from behind the traveler, over the left shoulder. If the traveler is to cross the same street in the opposite direction, the near lane parallel traffic comes from across the perpendicular street (in front). The traveler needs to learn to distinguish these two crossing scenarios. Finally, a surge in O&M refers to the visual and auditory signals made by a previously stopped car that starts to move. Because in most circumstances, the NLPTS corresponds to the change of the traffic light to green and coincides with the onset of the WALK sign, learning to locate the near lane parallel traffic and to detect its surge enables a traveler to make correct crossing decision even if he cannot see the pedestrian signal.

However, in cities such as Birmingham, Alabama, cars in the parallel traffic are allowed to make right turns when the traffic light is red. This weakens the salience of the NLPTS. If the surge made by such a turning car is used to time crossing, the traveler may risk being run over by the turning car or end up in the moving perpendicular traffic. Therefore, the correct use of NLPTS is not to start crossing immediately when a surge is detected, but to wait until one of the cars in the near lane entering the center of the street intersection and thus to confirm that the surge is indeed a NLPTS. This strategy sacrifices a few precious seconds at the beginning of the pedestrian phase to improve the safety of crossing decision. The NLPTS skills were taught to almost all individuals seeking O&M training, including totally blind individuals.

### Study environments

#### Real streets

Multiple real street intersections on the University of Alabama at Birmingham campus were selected for both road safety evaluations and NLPTS skill training. These are typical urban center cross intersections with different number of lanes. The traffic flow during the day was from 150 to 1300 cars/hour and the duration of the pedestrian phase was between 27 and 74 seconds, because of the differences between arterial and collector roads.

#### Virtual streets

**Fig 2:**
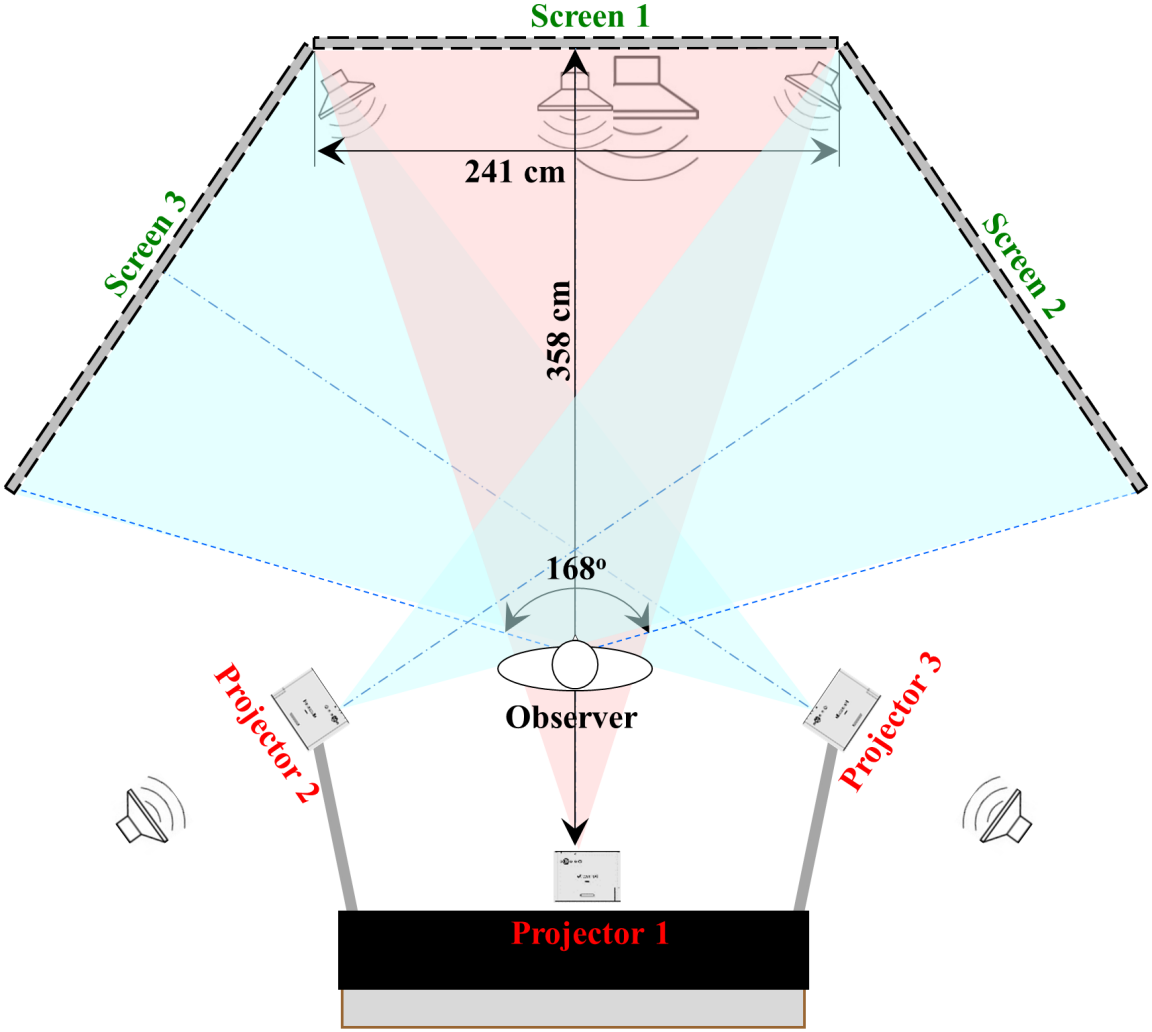
The visual and auditory display components of the VR simulation used in the research.

The VR simulator used in this research consisted of a game computer that generate real-time street intersection scenarios, three XVGA projectors (Panasonic PT-LB90NTU), three 241 x 183 cm screens and a 5.1 surrounding sound system (Logitech Z90). For an observe standing 3 m in front of the center screen, the display presented a 168° x 35° field of view (Fig 2). The hardware of the simulator is quite affordable ($5K-6K). No head or body position sensors were used to update the display. This projection VR allowed direct communication between the COMS and the participant during training. It also minimized the risk of cybersickness that happens often in head-mount display VR. This is important because the majority of individuals with low vision are also old and it is known that this population has balance problems and a higher risk to fall [28–30].

Virtual content development platform 3DVIA virTools (Dassault Systems) was used to build 3D models of the street intersections and the dynamic elements such as cars and pedestrians. Four real street intersections around the University of Alabama at Birmingham campus were chosen as the models for constructing virtual intersections. They differed in traffic patterns (one-way and two-way), street configuration (with and without central refuge) and surroundings (with and without overpass). Geographic Information System maps, Google Street images and photos taken by us provided the information to build the buildings around the intersections and the traffic control elements. Video recordings from the streets and vehicle count data were used to characterize the traffic flow.

**Fig 3:**
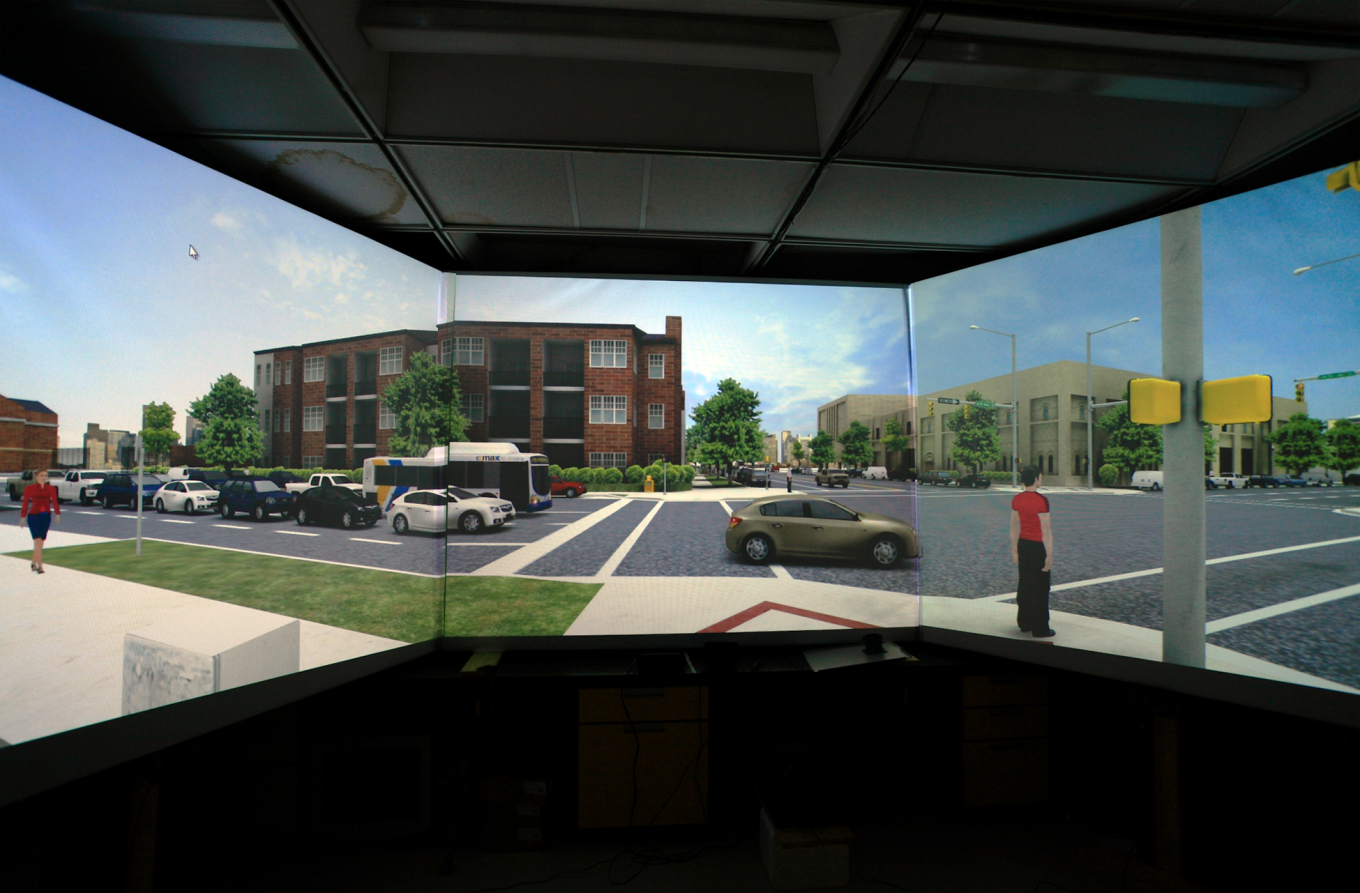
A virtual street intersection viewed from the location of a participant.

Once started, the simulator ran continuously, randomly generating and displaying dynamic elements of the street intersections. Traffic and pedestrian control signal cycles are built to simulate the real street intersections. The movements of cars and pedestrians were synchronized with traffic control signals and obeyed traffic laws. Cars stayed in lanes, blinked turning light and gave way to pedestrians when turning. The default setting simulated mid-day traffic after the morning rush-hour. The lighting was that of clear mid-day without strong oblique light and strong shadows. Only faint shadows could be seen under the cars. The foliage was that of late spring. Each car had an engine noise source under the hood to provide the traffic noise. A white noise was used to simulate street background noise recorded from the streets. There was no interaction between the participant and the simulator display. The participant basically viewed a computer-generated wide-screen movie. Fig 3 shows a virtual street intersection viewed from the location of a participant. A video clip showing two of the virtual street intersections and the real street intersections they were modeled after can be found in S1 Video.

Each virtual street intersection was a 3D model of buildings, streets, cars and pedestrians. The model was viewed by a cluster of three virtual cameras whose configuration matched the physical layout of the three projection screens that the participant viewed. Three virtual microphones at the same location as the virtual camera collected sound signals from the sound field made by the moving cars and fed them to the surrounding sound system that the participant heard. Moving the cameras and microphones to different locations in the intersection model allowed us to configure different street crossing scenarios using computer keyboard and mouse. Once a scenario was set, it was saved to a dropdown list and could be used repeatedly. A typical crossing scenario simulated a person standing at the curb side, facing the crosswalk of the perpendicular street, with one block of the traffic on both sides. The default traffic setting was 30 cars/min and 20 pedestrians/min in the intersection. The COMS could adjust the number of cars and pedestrians independently to facilitate training.

### Procedures

#### Safety evaluation protocol

The protocols for the pre- and post-training real street evaluation sessions were identical. The low vision participant was sight-guided to one of the pre-determined real street corners by a COMS and was positioned facing away from the perpendicular street. During the DON’T WALK phase, the participant was turned around to face the crosswalk of the perpendicular street and was instructed to say “GO” at the moment he thought was the safest time to cross the street. The participant was then turned to face away from the perpendicular street until the next traffic signal cycle. No walking into the street was allowed. A trial like this was repeated three time at each crossing scenario before the participant was walked to the another evaluation scenario. The participant was evaluated at 4 crossing scenarios in each evaluation session. The experimenters offered no comments, suggestions or feedbacks on the participant’s performance during evaluation.

**Fig 4:**
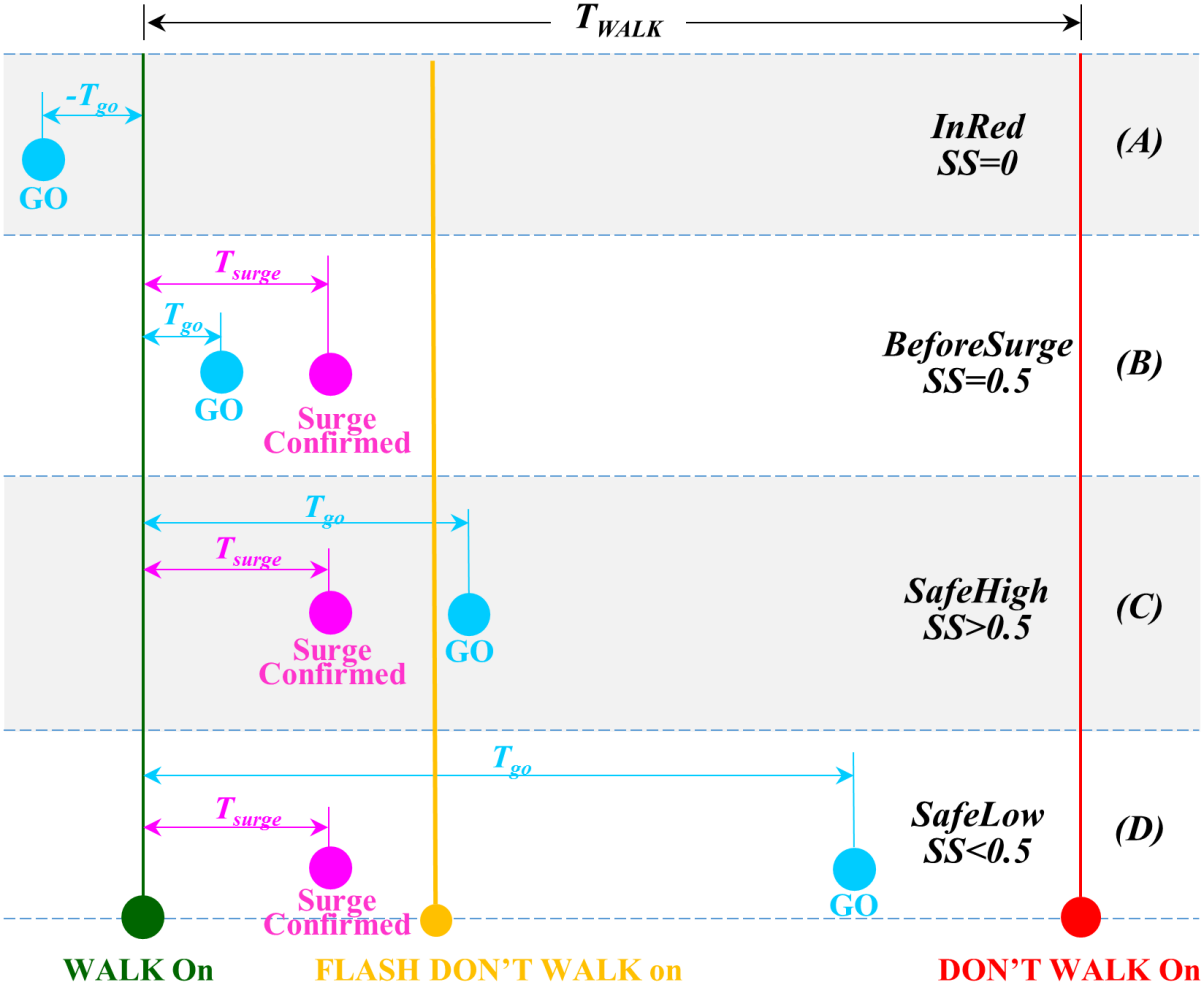
Events and time intervals used to evaluate safety of street crossing timing. Four possible combinations of the participant’s GO call (cyan circles), the onset of the WALK sign (the green circle) and the time when a near lane parallel traffic surge being confirmed (magenta circles) are shown in a WALK phase delineated by the onset of the WALK sign (WALK On) and the onset of the DON’T WALK sign (DON’T WALK On). (A-D) illustrate the four possible time sequences of the WALK On, Surge Confirmed and GO events.

During each trial, the times of three events were recorded using a stopwatch by the experimenters. 1) The time when the WALK sign was turned on (green circle in Fig 4). The sighted experimenters could see this event but the low vision participant could not. 2) The time when the rear bumper of the first straight-going car passed the outmost boundary of the crosswalk (magenta circles in Fig 4). This criterion was used by the sighted experimenters to produce consistent physical measurements of a car entering the center of the intersection. The low vision participant might be able to see a car moving into the intersection, but were not taught and might not have the vision to use this criterion. 3) The time when the participant said GO (cyan circles in Fig 4). The time interval between the first two times was designated *T*_*surge*_, representing the time taken to establish a true NLPTS. The time interval between the first and the third times was designated *T*_*go*_, representing the time taken for the participant to decide to start crossing the street. The duration of the pedestrian phase of the crossing scenario, *T*_*WALK*_, was also recorded. Fig 4 shows four possible sequences of the three events that have different safety consequences. (A) The participant said GO before the onset of the WALK sign. The time between the GO call and the onset of WALK sign in seconds was recorded as a negative number. The NLPTS event was irrelevant here. This decision was totally unsafe because the perpendicular traffic was running when the WALK sign was not on and the traveler would walk right into it. This was categorized as InRed. (B) The participant said GO after the onset of the WALK sign but before a NLPTS was established. *T*_*go*_ was shorter than *T*_*surge*_ in this case. Both were recorded as positive numbers in seconds. The safety of this decision was uncertain, depending on whether the surge was made by a turning car or a straight-going car. It was categorized as BeforeSurge. (C) The participant said GO after the onset of WALK sign and shortly after NLPTS was confirmed. The safety level of this decision was high because the traveler had a large portion of the *T*_*WALK*_ duration to walk across the street. It was categorized as SafeHigh. (D) The participant said GO after the onset of WALK and after NLPTS was confirmed but was near the end of the pedestrian phase. The safety level of this decision was lower because there was only a small portion of the *T*_*WALK*_ duration left and the traveler might not have enough time to walk across the street. It was categorized as SafeLow. Finally, the participant could have said GO after the current pedestrian phase was over and the next DON’T WALK phase began, but this had not happened in this research.

In each evaluation section, two experimenters were making independent measurement of *T*_*go*_ and *T*_*surge*_. Because the measurements were mere recording of physical events, the inter-rater agreements were high. The Pearson r’s for *T*_*go*_ and *T*_*surge*_ were 0.992 and 0.922, respectively (p < 0.0005). The mean *T*_*surge*_ measures from the two experimenters were 3.94 and 4.21 sec. The mean *T*_*go*_ measures were 7.24 and 7.34 sec. Both differences were not significant (p = 0.217 and 0.471).

#### Safety score of crossing decision

While *T*_*go*_ was a direct measure of the participant’s crossing timing and a shorter *T*_*go*_ usually indicated a safer decision, there were difficulties to quantify the safety of the timing using raw *T*_*go*_ data. First, whether a *T*_*go*_ was safe or not depended on the total duration of the pedestrian phase, *T*_*WALK*_. For example, a 10 seconds *T*_*go*_ is probably not quite safe in a crossing scenario where the *T*_*WALK*_ is 27 seconds because there are only 17 seconds left for the traveler to walk across the street. However, the same *T*_*go*_ becomes much saver in a scenario with a *T*_*WALK*_ of 57 seconds because the traveler now has 47 seconds to walk across the street. Second, for the reason explained above, it was difficult to compare *T*_*go*_ values obtained from crossing scenarios with different *T*_*WALK*_ durations, which varied between 27 and 74 seconds among our scenarios. Third, as explained before, a *T*_*go*_ shorter than *T*_*surge*_ suggests failing to use the NLPTS correctly and thus may not be safe. For these reasons, the *T*_*go*_ data was converted into a safety score (SS) using the following equation:

$$Safety\;Score=\{\begin{array}{cc} 0 & T_{go}<0\\ 0.5 & 0\leq T_{go}<T_{surge}\\ \left(T_{walk}-T_{go}\right)/T_{walk} & T_{go}\geq T_{surge} \end{array}$$ (1)

The SS was a unit-less number between 0 and a value slightly smaller than 1.0. A large SS indicated a safer decision. If *T*_*go*_ was recorded as a negative number (Fig 4A), SS was zero because the participant said GO during the DON’T WALK period and was thus totally unsafe. If *T*_*go*_ was shorter than *T*_*surge*_ (Fig 4B), the safety of the participant’s decision was uncertain. He could be safe if the surge he detected was caused by the straight-going car but he could be unsafe if the surge was caused by a turning car. A SS of 0.5 was given to such a decision. If *T*_*go*_ was longer than *T*_*surge*_ (Fig 4C and 4D), the crossing decision was made within the pedestrian phase, and the SS value was the proportion of the *T*_*WALK*_ duration left after the participant said GO. Notice that SS decreased linearly with increasing length of *T*_*go*_ in this case. This was because the longer was the *T*_*go*_ delay, the shorter time left in the pedestrian phase for the participant to walk across the street. Also notice that the SS could not reach 1.0 because a low vision participant could not see the onset of the WALK sign and immediately give a correct GO call. Instead he had to wait for a NLPTS to be established. Although he could not use the entire *T*_*WALK*_, he could make use of a very substantial portion of it, if he was proficient with the NLPTS skill.

#### NLPTS skill training protocols for virtual and real street

After the pre-training evaluation, the participant of the virtual street training group was taught the NLPTS skills in virtual streets by a COMS. The lights of the simulator room were turned off so that the projected image on the screens provided the only lighting. This greatly increased the contrast of the visual display. When the COMS needed to change the crossing scenario, the participant was asked to close his eyes to prevent him from getting sick by the fast whole field movement. The participant was allowed to use the eye wear and hearing aids that they routinely use and was allowed to hold his white cane if that made him feeling more comfortable.

The participant was asked to stand in the center of the simulator, approximately three meters in front of the center screen. A chair was placed in front of the participant, the back of which was set parallel to the perpendicular street of the training session. The participant was asked to hold the back of the chair to orient himself, to provide some support and to resume the correct orientation after taking a break. The participant was provided with another chair to sit in during a break.

Using the traffic flow in the virtual streets, the COMS explained the concepts involved in NLPTS, the actions to be taken and the cautions to be exercised. She also provided instructions on how to use visual and auditory information to identify the elements of NLPTS and to detect one correctly. The following is a sample script that the COMS used to test Joe’s understanding of the concepts and to give feedbacks.

**COMS:** “Joe, please tell me where the parallel traffic lanes are.”

**COMS:** “Joe, please tell me where nearest lane is.”

**COMS:** “Joe, when the cars have a red light, where will they stop in this lane?”

**COMS:** “Joe, notice the cars going left and right have stopped.”

**COMS:** “Joe, can you tell me where the cars are waiting for the green light?”

**COMS:** “Joe, can you tell me when those cars drive through the intersection?”

**COMS:** “Good, that is when you cross the street. Say ‘Go’”

The COMS repeated these steps until the participant made correct responses and then switched to a different virtual street for practice. The training terminated when the COMS was certain that the participant had mastered the NLPTS skills. The length of the training depended on the severity of the participant’s sensory impairment, his ability to communicate with the COMS and his ability to follow instructions. The maximum training time in the VR for a participant was 30 minutes.

On the following visit to study site, usually the next day, the participant was given a review of the skills taught for a maximum of 15 minutes before he/she was given the post-training evaluation.

The same COMS used the same protocol to teach NLPTS skills in real streets. The participants of the RST group had to be sight-guided by the COMS to different training scenarios to ensure safety. No chairs provided for keeping orientation and rest.

### Participants

Low vision participants were recruited from the Center for Low Vision Rehabilitation of University of Alabama at Birmingham, Alabama Department of Rehabilitation Services and Alabama Institute for Deaf and Blind. The inclusion criteria included 19 years or older, having severe to profound vision loss that made it difficult to find or to use pedestrian signals reliably, having a demonstrated need for O&M services but had not previously received training in using the NLPTS, physically strong enough to walk for a few street blocks and having sufficient mental and communication capability to understand and follow instructions in spoken English. After the initial screening, a potential participant was taken to real street corners to verify inability to use the pedestrian signals. Those who were unable to correctly report the status of the pedestrian signal across the street in 4 street intersections were enrolled into the research. The research was approved by the Institutional Review Board of the University of Alabama at Birmingham, and was conducted in accordance with the Declaration of Helsinki. A written informed consent was obtained from each participant before any testing and training was conducted.

### Data analysis

The *T*_*surge*_ and *T*_*go*_ data collected from the streets were entered into an Excel data sheet that automatically computed SS according to the *T*_*WALK*_ of the scenarios. A sample data sheet is shown in Table 1. A repeated ANOVA with TRAINING (pre vs. post) as the within-subject variable and GROUP (virtual vs. real street training) as the between-subjects variable was performed on the SS to assess transferability of VR training.

**Table 1:** Sample pre- and post-training real street evaluation data sheets.

Pre-Training Evaluation					Post-Training Evaluation				
Scenario *(T*_*WALK*_*)*	Near Lane	*T*_*go*_	*T*_*surge*_	Safety Score	Scenario *(T*_*WALK*_*)*	Near Lane	*T*_*go*_	*T*_*surge*_	Safety Score
**1 (*27sec)***	***Behind***	3.32	4.92	0.5	**1 (*74sec)***	***Behind***	3.07	2.47	0.946
		-1.33	NA	0			5.33	3.09	0.906
		4.89	3.47	0.819			8.4	6.87	0.853
**2 (*74sec)***	***In Front***	-19.29	NA	0	**2 (*27sec)***	***In Front***	4.95	4.56	0.913
		-19.87	NA	0			4.23	3.64	0.926
		-18.11	NA	0			5.38	4.53	0.906
**3 (*54sec)***	***In Front***	-6.49	NA	0	**3 (4*6sec)***	***In Front***	4.15	3.38	0.91
		-28.38	NA	0			3.71	3.07	0.919
		-33.22	NA	0			6.13	3.65	0.866
**4 (*54sec)***	***Behind***	-4.71	NA	0	**4 (*46sec)***	***Behind***	3.45	2.51	0.925
		4.97	2.53	0.908			5.04	3.13	0.89
		-19.21	NA	0			4.43	2.61	0.904
**Mean**				**0.186**	**Mean**				**0.905**
**SD**				**0.348**	**SD**				**0.026**

Participant #8: 55 years old; Male; African-American; Group assignment: VST

Scenario: Real street crossing scenarios used for pre- and post-training safety evaluations. The durations of the pedestrian phase of the scenarios in seconds (*T*_*WALK*_) are include in parentheses.

Near Lane: *Behind* means the near lane traffic is from behind (over the left shoulder). *In Front* means the near lane traffic is from front (across the perpendicular street).

*T*_*surge*_: NA means not available, happened when the participant said GO before the onset of the WALK sign.

## Results

### Participants

Twelve qualified low vision participants were enrolled into the research (Table 2). The ages of the participants ranged from 19 to 69 and the durations of visual impairment ranged from 0.5 to 36 years. The causes of visual impairment included retinitis pigmentosa, diabetic retinopathy, degenerative myopia, optic atrophy, Best disease, Usher’s disease and non-arteritic anterior ischemic optic neuropathy. One participant had mild hearing impairment. Another participant had severe hearing aid but wore habitual hearing aid. All participants were unable to use pedestrian signals reliably and all did not know the NLPTS skill prior to training, as determined in real streets.

**Table 2:** Description of the 12 low vision participants.

ID	Group	Age	Diagnoses	Sex	Race	Years DX	Visual Acuity
1	VST	69	Diabetic Retinopathy	F	AA	5	OD: HM
							OS: 20/607
2	VST	19	Retinitis Pigmentosa	M	W	5	OU: 20/3000
3	RST	57	Best Disease	F	W	2	OD: 20/320
							OS: 20/800
4	VST	51	Retinitis Pigmentosa	F	W	36	OU: 20/600
5	VST	26	Diabetic Retinopathy	F	AA	2	OD: 20/697
							OS: LP
6	RST	35	Optic Atrophy	F	AA	20	OD: 20/533
							OS: 20/1000
7	VST	47	Ushers & Retinitis Pigmentosa	M	W	24	OU: 20/800
8	VST	55	Degenerative Myopia	M	AA	35	OD: 20/1928
							OS: 20/1162
9	RST	52	Myopic Choroidal Degeneration	F	AA	15	OD: 20/1754
							OS: 20/400
10	RST	48	Retinitis Pigmentosa	F	AA	31	OD: LP
							OS: 20/2847
11	VST	54	OD: NAION	F	W	2	OD: 20/877
			OS: Trauma				OS: LP
12	VST	36	Diabetic Retinopathy	F	W	5	OD: 20/439
							OS: NLP

Group: Training group assignment (VST: virtual street training; RST: real street training)

Years DX: Years since diagnosis

NAION: Non-Arteritic Anterior Ischemic Optic Neuropathy

AA: African-American; W: White

OD: Right eye; OS: Left eye

HM: Hand motion; LP: Light perception; NLP: No light perception

The mean ages of the real and virtual street training groups were 48±9.4 and 45±16.5, respectively. The age difference was not significant (t_10_ = 0.45, p = 0.331).

### Crossing decision safety

**Fig 5:**
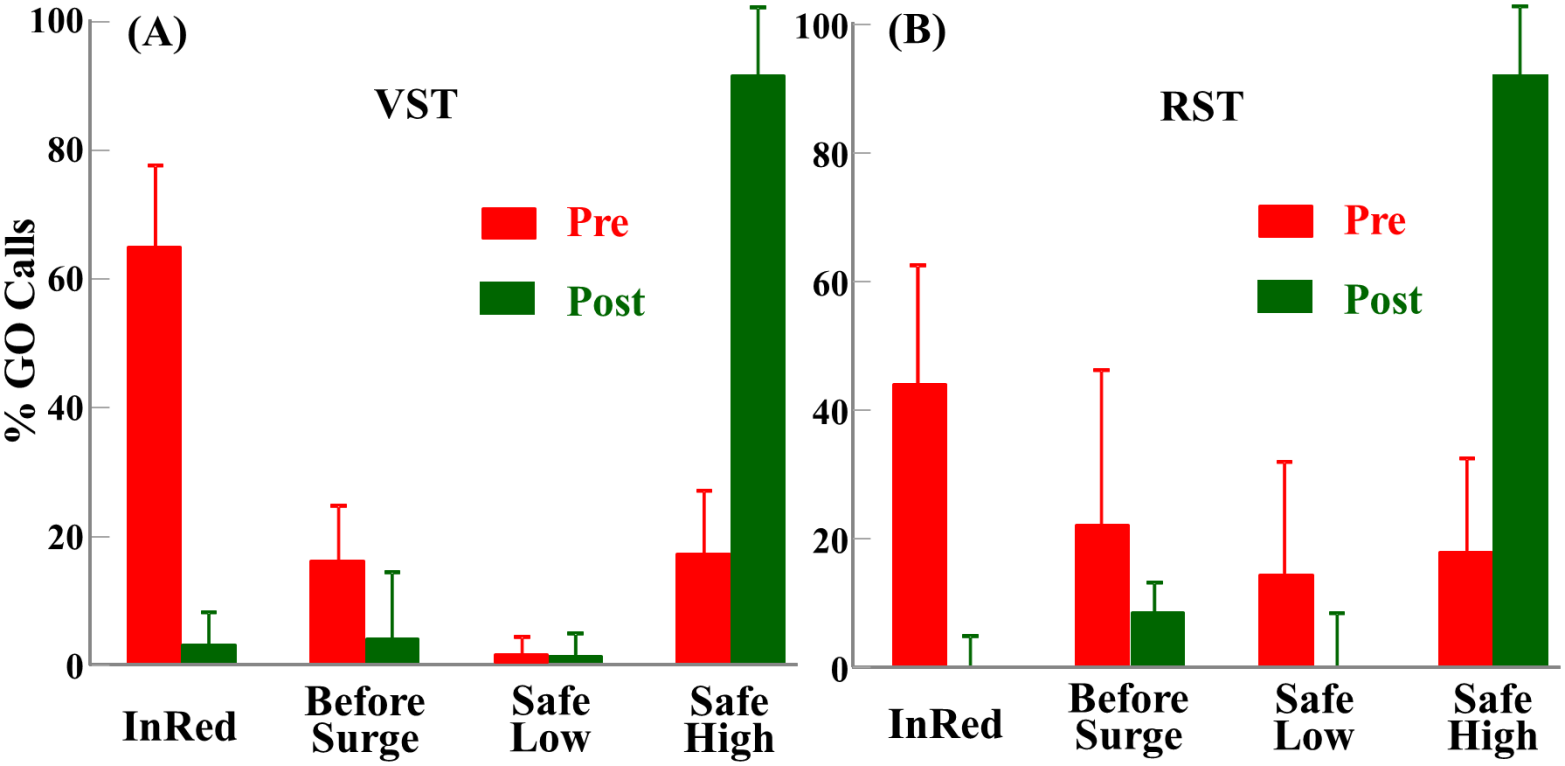
Pre- and Post-training safety categorization distributions for the virtual street and real street training groups. (A) Proportion of the GO calls falling into the InRed, BeforeSurge, SafeLow and SafeHigh categories obtained from the pre- (red bars) and post-training (green bars) evaluations for the virtual street training (VST) group. (B) Same distribution plots for the real street training (RST) group.

Fig 5 shows the proportions of the GO calls falling into the InRed, BeforeSurge, SafeLow and SafeHigh categories obtained from the pre-training (red bars) and post-training (green bars) evaluations for the virtual street training (A) and the real street training (B) groups. According to Eq 1, the safety score was 0 for the InRed category and 0.5 for the BeforeSurge category. The SafeLow category was defined as the participant saying “GO” in the latter half of the WALK phase (low safety, SS < 0.5, might not have enough time to walk across the street) and the SafeHigh category was defined as the participant saying “GO” shortly after confirming the NLPTS, in the first half of the WALK phase (high safety, SS > 0.5, had more time to walk across).

Prior to the NLPTS training, all participants showed highly unsafe behaviors in the pre-training real street evaluation. On average, the 8 participants of the virtual street training group said “GO” during the DON’T WALK phase 64% of the time (red “InRed” bar in Fig 5A) and said “GO” before a NLPTS was confirmed 17.0% of the time (red “BeforeSurge bar). They said “GO” in the latter half of the pedestrian phase 2% of the time (red SafeLow bar). In only 17% of the time their timing offered them more than 50% of the pedestrian phase for crossing (red SafeHigh bar). The primary cause of their unsafe timing appeared to be using the “gaps” in the perpendicular traffic (temporary absence of moving cars near the intersection on the perpendicular street). The participants also tried to use other tactics, such as observing other pedestrians (not advised). Similar distribution was found in the real street training group (red bars in Fig 5B). There was no significant difference in safety scores in pre-training evaluation between the two groups (F_1,10_ = 1.921, p = 0.196).

After the NLPTS training, the safety of all participants’ crossing decision timing was greatly improved. During post-training evaluations on real streets, participants in the virtual street training group said “GO” 3.0% of the time during the DON’T WALK phase, 4% of the time before surge and 1% of the time too late in the pedestrian phase (green “InRed”, “BeforeSurge” and “SafeLow” bars in Fig 5A). They said “GO” a few seconds after the surge was confirmed in 92% of the time, which allowed them an average 88.9% of the pedestrian phase for crossing. A similar pattern was also observed in the RST group (green bars in Fig 5B). There was no significant difference in safety scores in post-training evaluation between the two groups of participants (F_1,10_ = 0.006, p = 0.939). These results demonstrated a drastic shift of crossing decision safety from very unsafe to very safe after both virtual street and real street NLPTS skill training.

### Transferability of VR O&M training

Prior to training, the mean SS of the virtual and real street training groups were 0.20±0.11 and 0.31±0.15, respectively. The difference was not significant (F_1,10_ = 1.921, p = 0.196). After training, the mean SS of virtual and real street training groups were 0.821±0.083 and 0.817±0.090, respectively. The difference was not significant (F_1,10_ = 0.006, p = 0.939).

On average, the time needed to physically confirm a NLPTS (first straight-going car entering the center of the intersection), *T*_*surge*_, was 3.65±1.68 seconds, which was 8.46% of the total pedestrian phase duration (*T*_*WALK*_). After training, the participants said GO 5.43±2.95 seconds after a NLPTS was confirmed, which was 12.5% of *T*_*WALK*_. These left the participants more than 80% of the *T*_*WALK*_ duration to cross the street.

**Fig 6:**
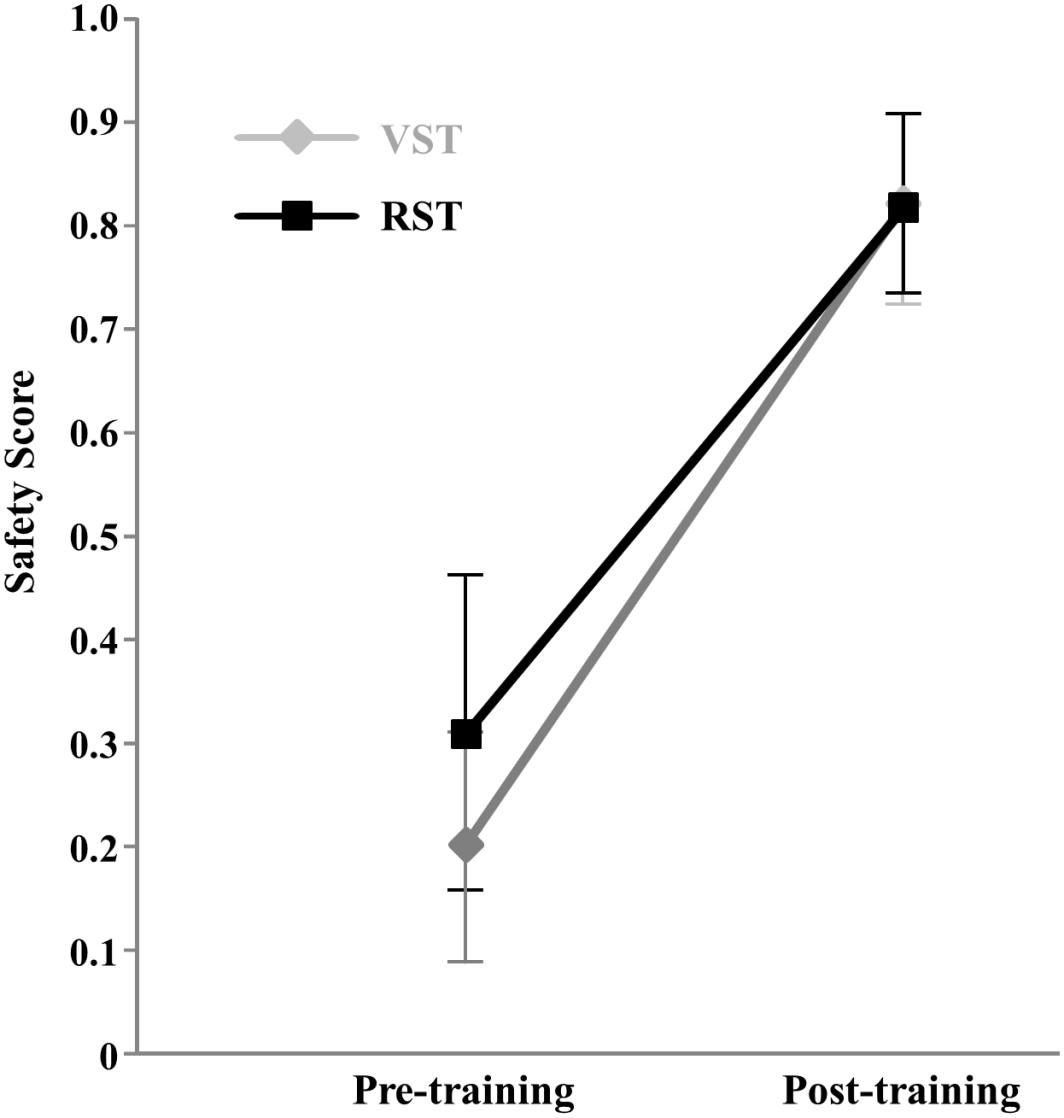
Pre- and post-training safety scores of the virtual street training (VST) and real street training (RST) groups.

A repeated measures ANOVA on the safety score was used assess the effectiveness of VR training (Fig 6). There was a highly significant TRAINING effect (F_1,10_ = 288.3, p<0.0005). The GROUP effect was not significant (F_1,10_ = 0.821, p = 0.386), and the TRAINING*GROUP interaction was not significant (F_1,10_ = 2.80, p = 0.125). This analysis demonstrated that both the virtual and real street training groups made safer real street crossing decisions after the NLPTS training and that there was no difference between the groups in terms of training benefits. In other words, low vision participants could transfer their VR-acquired skills to real streets and virtual street training was as efficient as real street training.

## Discussion

In this research, we demonstrate a strong positive transfer of VR-trained skill to real streets. Low vision participants, whose vision was too poor to use pedestrian signs, learned a set O&M skill in virtual streets and successfully used them to improve their street crossing timing in real streets (Fig 5). The training effect obtained from the virtual street training was comparable to that from the real street training (Fig 6). The high rate of positive transfer of VR-trained NLPTS skills to real streets also demonstrated that our low-cost simulator was adequate in training these O&M skills. As far as we know, this is the first time a positive transfer of VR-trained O&M skills being demonstrated in individuals with impaired but usable vision.

After training, a few participants volunteered some comments about the VR training. They appeared to like the virtual streets. “I liked that I was standing on a street corner and no cars would hit me.” “It kinda made me aware of my surroundings in all four directions. … I really thought the simulator was good.” The participants considered the VR training they received helpful. “It (VR) showed me something that I was not aware of. It prepared me for the outside.” “It (VR) encouraged me to learn more travel skills. I know I can travel independently. I felt the VR simulator was helpful.” Our participants also pointed out some shortcomings. “It made me nervous to learn something new. It was a crazy intersection.” Future development of clinical deployable VR applications should include subjective usability as an outcome measure.

### NLPTS and traveler’s ability to cross the street

The safety score (SS) was a measure of how promptly a traveler started crossing after the pedestrian sign turned to WALK. Does a good SS (close to 1.0) guarantee the traveler a safe crossing? After the NLPTS training, our participants made “GO” calls at an average of 5.55±3.02 sec after the onset of the WALK sign, indicating that they possessed good skills to start crossing early, within the 7-sec WALK period. However, whether they could make to the far side of the street depended whether they could walk 3.5 feet/sec or faster [27]. Three of our participants told us how fast they thought they could walk and their reasoning. “I take my time so I can feel the surface so I don’t fall. About 2 feet per second.” (participant #4) “If it’s well light I’m a fast walker. Five feet per second, I walk fast.” (participant #6) “I’m tall and I have really long legs, but I move slow because I don’t want to run into a pole or fall or anything. If I know where I’m going, maybe 3 feet per second.” (participant #8) Despite the large differences among their perceived walking speeds, an ANOVA showed no significant differences among their safety scores (F_2,33_ = 0.572, p = 0.570). This should not be surprising because the NLPTS training they received concerned only about detecting the onset of the WALK sign (using NLPTS as a surrogate). If they learned the skills well, their SS should not be affected by their perceived walking speed. However, if the participants’ perceived walking speeds turned out to be equal to their actual walking speeds, then participant #4 (2 feet/sec) would not be safe to cross any streets. Participant #6 (3 feet/sec) might be able to reach the far side of some streets and participant #8 (5 feet/sec) was likely to make safe crossing on any streets. Does this large variation in participants’ ability to cross streets negate the usefulness of NLPTS training? Not at all. NLPTS only teaches the traveler how to start crossing early, and as shown by our results, this training was successful. To achieve safe crossing of signal controlled streets, the traveler also need to learn how to walk fast with impaired vision and how to judge whether a street is crossable, but these are totally different skills from the NLPTS.

### VR training in O&M rehabilitation

The role of VR training in O&M rehabilitation is to prepare low vision trainees in a safe and efficient training environment. Like in many other fields of skill acquisition, such as pilot and surgical trainings, where errors in real world incur tremendous human and economic costs, VR is not meant to replace practicing flying a real plane, operating on a real patient or navigating real streets but it can teach many necessary knowledge and skills with minimal risk, less cost and higher efficiency so that the trainees can apply the knowledge and skills in real world tasks instead of learning everything from scratch in the real world. Our study has shown that VR-learned O&M skills can indeed be applied to real world.

There are many skills in the O&M curriculum that can be trained efficiently in VR, but there are others, such as white cane use and veering correction, that are impossible or difficult to train in an affordable VR, and thus have to be trained in real streets. A well-thought-out O&M curriculum that integrate VR training with real street training may bring welcome changes to the O&M profession in several ways. First, in the safe virtual street, the safeguarding role of the COMS is greatly reduced. If future development of VR-based courseware can also take up of the teaching role, the scarce and costly COMS resource can be focused more on trainings that have to be done on real street, and thus improves the accessibility and affordability of O&M rehabilitation. Second, the man-made safe VR is a perfect platform to implement some the proven learning theories. Structured courseware with stepwise progression of task difficulty and scenario complexity, objective performance metrics, proximal feedback, repeated practice and learning from mistakes can be developed to make training more efficient. Third, the VR technology makes it possible to provide O&M rehabilitation through networked satellite stations so that low vision trainees can learn O&M skills at their convenient times and locations.

### Limitations

Several limitations of the current research were noticed.

The research was designed to compared the new, virtual street O&M training and the standard of care, real street training. If virtual street training could result in a comparable gain in real street safety, as compared to the standard of care, then the ground is laid for future exploration of VR technology’s potentials in improving the accessibility, affordability and efficiency of O&M rehabilitation. One limitation of this design is the lack of a non-treatment control arm, which can serve to control the effect of the initial evaluation. Our decision to use the limited number of available low vision participants in a standard of care control group instead of a non-treatment control was based on the following considerations. 1) The NLPTS skills were the most frequently taught skills to low vision patients, indicating that these skills might not be easily picked up spontaneously. Normally-sighted individuals use more direct and more salient signals, the traffic signal change, to guide their decision and would not routinely use a surrogate, such as the NLPTS. 2) The participants received absolutely no instruction or hint on how to determine the timing of crossing. They were given no feedbacks as to how well they did either. While the exposure to the street crossing task might affect the participants in a positive way, such as wakening some past street crossing experience, it was unlikely that the effect would result in systematic improvement of crossing behavior, because the basis for their previous street navigation experiences, good vision, was not available. 3) The fact that our participants, who had severely impaired vision for years, even decades, before enrolling into the study, did very poorly in the pre-training evaluation suggested also suggested that the specific training of crossing skills, not just exposures, was needed to make safe crossing decisions. In hindsight, the very short time intervals between the confirmation of NLPTS and the participants’ GO calls observed in the post-training evaluation suggested that it was the NLPTS skills that the participants learned during training was used in their crossing decision making. Nevertheless, a non-treatment control would have provided unequivocal evidence about the effect of pre-training evaluation, and future studies should take this into consideration.

As discussed above, our simulator simulated only a subset of the sensory cues of the real world and some of our experimental conditions, for example, turning off the lights in the VR room and providing a chair for assistance, did not match those of the real streets. These need to be taken into consideration when evaluating the study results. For example, we don’t know whether an ambient light level that matches that of the real street has an effect on training outcome. One important issue in the future development of VR-based O&M training protocols is the trade-off between high simulation fidelity (higher cost) and training efficiency.

This study included only adult participants. There are greater physical, psychological and socioeconomic benefits to conduct O&M training in children and young adults with early-onset visual impairments. However, the lack of prior knowledge of road safety rules, immature cognitive development and the short attention span of this population can pose special challenges to O&M training. The VR training protocol we used may not be adequate for this population.

The NLPTS skills were taught by a COMS in both the virtual and real streets. There was no interaction between the simulator and student. The COMS had to collect student responses to questions and instructions to gauge the student’s proficiency in using the skills. This was because the purpose of the research was to determine the feasibility of VR as a sensory platform for O&M training. Now that the feasibility has been established, the future research and development will be focus on adding sensors to collect the student’s responses and develop interactive, game-like training routines to automate the instruction and practice process and to minimize the intervention of the COMS.

Participants in this study had severe to profound visual impairment. Although our simulation appeared to be adequate for training this sample of participants, it may be inadequate for others. Individuals with better vision may require higher visual display quality from the simulator. Individuals with poorer vision may require better auditory display than the 5.1 surround sound used in this study. Matching display quality, skills taught and patient’s visual capacity will be the first issues to consider to design future VR training protocols.

Our post-training evaluation was conducted a few days after VR training. It thus reflected short-term training gain. Due to the logistical difficulties in low vision participant retention, the long-term retainment of the VR-trained skills was not evaluated. Although real street trained O&M skills usually retain well unless there is a significant decay of vision, long-term retainment of VR-trained skills is unknown. This aspect of VR training should be considered in future studies.

## Conclusions

This research study demonstrated that O&M skills learned in virtual streets can be used to improve real streets safety in individuals with low vision. Moreover, the training benefit from virtual streets can be as large as that from real street. Strong evidence thus exists that a computer generated virtual environment can be a viable platform for low vision O&M training.

## Supporting information

S1 Video Video of virtual and real street intersections. (MP4)

S1 Table Data from all participants. (DOCX)

## Abbreviations

COMS: certified O&M specialist
NLPTS: near lane parallel traffic surge
O&M: Orientation and Mobility
SS: safety score
VR: virtual reality
