# Supplementary material for: Individuals with severely impaired vision can learn useful orientation and mobility skills in virtual streets and can use them to improve real street safety
Source: PLoS One. 2017 Apr 26;12(4):e0176534. doi: 10.1371/journal.pone.0176534 (PMC5405961; doi:10.1371/journal.pone.0176534)
Supplement: S1 Table — (DOCX) [file pone.0176534.s002.docx]

**Supporting Information File –Data from all participants**

| **ID: 03** |  |  |  |  |  |  |  |  |  |
| --- | --- | --- | --- | --- | --- | --- | --- | --- | --- |
| **Pre-Training Evaluation** | | | | | **Post-Training Evaluation** | | | | |
| **Scenario** | **NearLane** | **Tgo** | **Surge** | **Safety** | **Scenario** | **NearLane** | **Tgo** | **Surge** | **Safety** |
| **1** | behind | 2.46 | 19.69 | **0.500** | **1** | behind | 3.87 | 2.39 | **0.948** |
|  |  | -1.93 | NA | **0.000** |  |  | 4.35 | 2.91 | **0.941** |
|  |  | -16.05 | NA | **0.000** |  |  | 4.73 | 3.92 | **0.936** |
| **2** | in front | -2.18 | NA | **0.000** | **2** | in front | 1.62 | 1.15 | **0.940** |
|  |  | -4.21 | NA | **0.000** |  |  | 4.7 | 2.59 | **0.826** |
|  |  | -8.96 | NA | **0.000** |  |  | 4.58 | 3.97 | **0.830** |
| **3** | in front | -2.7 | NA | **0.000** | **3** | in front | 7.16 | 6.23 | **0.844** |
|  |  | 5.81 | 3.15 | **0.892** |  |  | 9.08 | 8.03 | **0.803** |
|  |  | -1.78 | NA | **0.000** |  |  | 3.22 | 2.54 | **0.930** |
| **4** | behind | -4.38 | NA | **0.000** | **4** | behind | 3.87 | 5.4 | **0.500** |
|  |  | 13.22 | 4.34 | **0.755** |  |  | 4.05 | 4.34 | **0.500** |
|  |  | 14.47 | 3.58 | **0.732** |  |  | 4.43 | 5.83 | **0.500** |
|  |  |  |  |  |  |  |  |  |  |
| **Mean** |  |  |  | **0.240** |  |  |  |  | **0.792** |
| **Stdev** |  |  |  | **0.365** |  |  |  |  | **0.183** |
|  |  |  |  |  |  |  |  |  |  |
| **ID: 08** |  |  |  |  |  |  |  |  |  |
| **Pre-Training Evaluation** | | | | | **Post-Training Evaluation** | | | | |
| **Scenario** | **NearLane** | **Tgo** | **Surge** | **Safety** | **Scenario** | **NearLane** | **Tgo** | **Surge** | **Safety** |
| **1** | behind | 3.32 | 4.92 | **0.500** | **1** | behind | 3.07 | 2.47 | **0.959** |
|  |  | -1.33 | NA | **0.000** |  |  | 5.33 | 3.09 | **0.928** |
|  |  | 4.89 | 3.47 | **0.819** |  |  | 8.4 | 6.87 | **0.886** |
| **2** | in front | -19.29 | NA | **0.000** | **2** | in front | 4.95 | 4.56 | **0.817** |
|  |  | -19.87 | NA | **0.000** |  |  | 4.23 | 3.64 | **0.843** |
|  |  | -18.11 | NA | **0.000** |  |  | 5.38 | 4.53 | **0.801** |
| **3** | in front | -6.49 | NA | **0.000** | **3** | in front | 4.15 | 3.38 | **0.910** |
|  |  | -28.38 | NA | **0.000** |  |  | 3.71 | 3.07 | **0.919** |
|  |  | -33.22 | NA | **0.000** |  |  | 6.15 | 3.65 | **0.866** |
| **4** | behind | -4.71 | NA | **0.000** | **4** | behind | 3.45 | 2.51 | **0.925** |
|  |  | 4.97 | 2.53 | **0.908** |  |  | 5.04 | 3.13 | **0.890** |
|  |  | -19.21 | NA | **0.000** |  |  | 4.43 | 2.61 | **0.904** |
|  |  |  |  |  |  |  |  |  |  |
| **Mean** |  |  |  | **0.186** |  |  |  |  | **0.887** |
| **Stdev** |  |  |  | **0.348** |  |  |  |  | **0.047** |
|  |  |  |  |  |  |  |  |  |  |
| **ID: 09** |  |  |  |  |  |  |  |  |  |
| **Pre-Training Evaluation** | | | | | **Post-Training Evaluation** | | | | |
| **Scenario** | **NearLane** | **Tgo** | **Surge** | **Safety** | **Scenario** | **NearLane** | **Tgo** | **Surge** | **Safety** |
| **1** | behind | -21.63 | NA | **0.000** | **1** | behind | 5.77 | 3.23 | **0.922** |
|  |  | 3.13 | 5.09 | **0.500** |  |  | 3.81 | 3.51 | **0.949** |
|  |  | -19.18 | NA | **0.000** |  |  | 6.21 | 3.54 | **0.916** |
|  |  | -28.55 | NA | **0.000** |  |  |  |  |  |
| **2** | in front | -15.08 | NA | **0.000** | **2** | in front | 3.84 | 3.29 | **0.858** |
|  |  | 1.64 | 6.15 | **0.500** |  |  | 3.87 | 2.28 | **0.857** |
|  |  | -6.93 | NA | **0.000** |  |  | 5.97 | 4.99 | **0.779** |
| **3** | in front | -1.49 | NA | **0.000** | **3** | in front | 4.81 | 3.62 | **0.895** |
|  |  | 2.53 | 3.93 | **0.500** |  |  | 6.66 | 3.83 | **0.855** |
|  |  | 2.73 | 3.67 | **0.500** |  |  | 8.5 | 5.91 | **0.815** |
| **4** | behind | 5.47 | 2.38 | **0.899** | **4** | behind | 22.34 | 7.47 | **0.514** |
|  |  | -11.26 | NA | **0.000** |  |  | 5.08 | 4.6 | **0.890** |
|  |  | 4.9 | 3.46 | **0.909** |  |  | NA | NA | **0.000** |
|  |  |  |  |  |  |  |  |  |  |
| **Mean** |  |  |  | **0.293** |  |  |  |  | **0.771** |
| **Stdev** |  |  |  | **0.356** |  |  |  |  | **0.268** |
|  |  |  |  |  |  |  |  |  |  |
| **ID: 10** |  |  |  |  |  |  |  |  |  |
| **Pre-Training Evaluation** | | | | | **Post-Training Evaluation** | | | | |
| **Scenario** | **NearLane** | **Tgo** | **Surge** | **Safety** | **Scenario** | **NearLane** | **Tgo** | **Surge** | **Safety** |
| **1** | behind | 5.38 | 1.92 | **0.801** | **1** | behind | NA | NA | **0.000** |
|  |  | 19.34 | 1.93 | **0.284** |  |  | 4.58 | 4.06 | **0.938** |
|  |  | 23.18 | 4.14 | **0.141** |  |  | 3.73 | 2.34 | **0.950** |
| **2** | in front | 3.53 | 4.46 | **0.500** | **2** | in front | 5.35 | 4.28 | **0.802** |
|  |  | 18.77 | 3.07 | **0.746** |  |  | 7.3 | 4.21 | **0.730** |
|  |  | -12.39 | NA | **0.000** |  |  | 5.43 | 4.28 | **0.799** |
| **3** | in front | 17.43 | 2.37 | **0.677** | **3** | in front | 10.27 | 3.82 | **0.777** |
|  |  | 12.65 | 2.54 | **0.766** |  |  | 3.63 | 4.35 | **0.500** |
|  |  | -2.61 | NA | **0.000** |  |  | 5.52 | 4.5 | **0.880** |
| **4** | behind | -14.7 | NA | **0.000** | **4** | behind | 6.7 | 4.84 | **0.854** |
|  |  | -3.98 | NA | **0.000** |  |  | 6.57 | 4.16 | **0.857** |
|  |  | 33.8 | 4.27 | **0.374** |  |  | 6.36 | 4.99 | **0.862** |
|  |  |  |  |  |  |  |  |  |  |
| **Mean** |  |  |  | **0.357** |  |  |  |  | **0.746** |
| **Stdev** |  |  |  | **0.330** |  |  |  |  | **0.262** |
|  |  |  |  |  |  |  |  |  |  |
| **ID: 01** |  |  |  |  |  |  |  |  |  |
| **Pre-Training Evaluation** | | | | | **Post-Training Evaluation** | | | | |
| **Scenario** | **NearLane** | **Tgo** | **Surge** | **Safety** | **Scenario** | **NearLane** | **Tgo** | **Surge** | **Safety** |
| **1** | behind | -13.75 | NA | **0.000** | **1** | behind | 4.59 | 4.12 | **0.938** |
|  |  | 2.29 | 8.73 | **0.500** |  |  | 6.65 | 4.83 | **0.910** |
|  |  | 1.59 | 13.4 | **0.500** |  |  | 4.62 | 2.96 | **0.938** |
| **2** | in front | -22.16 | NA | **0.000** | **2** | in front | 3.97 | 3.37 | **0.853** |
|  |  | 6.16 | 3.79 | **0.917** |  |  | 3.93 | 3.63 | **0.854** |
|  |  | -22.44 | NA | **0.000** |  |  | 4.4 | 3.37 | **0.837** |
| **3** | in front | 6.87 | 2.78 | **0.873** | **3** | in front | 6.67 | 5.65 | **0.855** |
|  |  | 11.42 | 4.34 | **0.789** |  |  | 2.27 | 1.37 | **0.951** |
|  |  | -1.52 | NA | **0.000** |  |  | 3.36 | 2.84 | **0.927** |
| **4** | behind | -0.72 | NA | **0.000** | **4** | behind | 3.74 | 3.32 | **0.919** |
|  |  | -13.52 | NA | **0.000** |  |  | 4.61 | 4.13 | **0.900** |
|  |  | 10.36 | 1.69 | **0.808** |  |  | 3.6 | 3.34 | **0.922** |
|  |  |  |  |  |  |  |  |  |  |
| **Mean** |  |  |  | **0.366** |  |  |  |  | **0.900** |
| **Stdev** |  |  |  | **0.402** |  |  |  |  | **0.040** |
|  |  |  |  |  |  |  |  |  |  |
| **ID: 02** |  |  |  |  |  |  |  |  |  |
| **Pre-Training Evaluation** | | | | | **Post-Training Evaluation** | | | | |
| **Scenario** | **NearLane** | **Tgo** | **Surge** | **Safety** | **Scenario** | **NearLane** | **Tgo** | **Surge** | **Safety** |
| **1** | behind | 29.14 | 5.8 | **-0.079** | **1** | behind | -5.87 | NA | **0.000** |
|  |  | 3.09 | 5.1 | **0.500** |  |  | 21.6 | 4.37 | **0.708** |
|  |  | 25.53 | 3.52 | **0.054** |  |  | 11.36 | 4.59 | **0.846** |
| **2** | in front | -12.63 | NA | **0.000** | **2** | in front | 4.05 | 3.66 | **0.850** |
|  |  | -13.18 | NA | **0.000** |  |  | 5.64 | 4.28 | **0.791** |
|  |  | NA | NA | **0.000** |  |  | 5.23 | 3.84 | **0.806** |
| **3** | in front | -11.64 | NA | **0.000** | **3** | in front | 9.69 | 2.32 | **0.789** |
|  |  | -5.37 | NA | **0.000** |  |  | 23.54 | 11.36 | **0.488** |
|  |  | -1.95 | NA | **0.000** |  |  | 8.62 | 4.53 | **0.813** |
| **4** | behind | 28.14 | 4.13 | **0.479** | **4** | behind | 9.22 | 3.28 | **0.800** |
|  |  | -26.97 | NA | **0.000** |  |  | 13.62 | 8.05 | **0.704** |
|  |  | -8.68 | NA | **0.000** |  |  | 7.78 | 2.46 | **0.831** |
|  |  |  |  |  |  |  |  |  |  |
| **Mean** |  |  |  | **0.080** |  |  |  |  | **0.702** |
| **Stdev** |  |  |  | **0.194** |  |  |  |  | **0.242** |
|  |  |  |  |  |  |  |  |  |  |
| **ID: 04** |  |  |  |  |  |  |  |  |  |
| **Pre-Training Evaluation** | | | | | **Post-Training Evaluation** | | | | |
| **Scenario** | **NearLane** | **Tgo** | **Surge** | **Safety** | **Scenario** | **NearLane** | **Tgo** | **Surge** | **Safety** |
| **1** | behind | 3.37 | 2.52 | **0.875** | **1** | behind | 3.49 | 2.56 | **0.953** |
|  |  | 1.67 | 7.33 | **0.500** |  |  | 2.8 | 2.37 | **0.962** |
|  |  | 1.35 | 4.17 | **0.500** |  |  | 5.43 | 3.07 | **0.927** |
| **2** | in front | -4.61 | NA | **0.000** | **2** | in front | 7.78 | 6.96 | **0.712** |
|  |  | 1.48 | 4.48 | **0.500** |  |  | 8.61 | 7.84 | **0.681** |
|  |  | 1.17 | 5.18 | **0.500** |  |  | 4.66 | 2.96 | **0.827** |
| **3** | in front | -6.93 | NA | **0.000** | **3** | in front | 5.6 | 4.75 | **0.878** |
|  |  | 3.18 | 4.33 | **0.500** |  |  | 4.68 | 3.53 | **0.898** |
|  |  | -3.07 | NA | **0.000** |  |  | 4.85 | 3.66 | **0.895** |
| **4** | behind | 9.1 | 3.29 | **0.831** | **4** | behind | 5.68 | 3.95 | **0.877** |
|  |  | 2.46 | 4.03 | **0.500** |  |  | 5.12 | 6.31 | **0.500** |
|  |  | 1.61 | 3.66 | **0.500** |  |  | 4.4 | 3.17 | **0.904** |
|  |  |  |  |  |  |  |  |  |  |
| **Mean** |  |  |  | **0.434** |  |  |  |  | **0.834** |
| **Stdev** |  |  |  | **0.294** |  |  |  |  | **0.137** |
|  |  |  |  |  |  |  |  |  |  |
| **ID: 05** |  |  |  |  |  |  |  |  |  |
| **Pre-Training Evaluation** | | | | | **Post-Training Evaluation** | | | | |
| **Scenario** | **NearLane** | **Tgo** | **Surge** | **Safety** | **Scenario** | **NearLane** | **Tgo** | **Surge** | **Safety** |
| **1** | behind | 15.4 | 5.2 | **0.430** | **1** | behind | 3.32 | 6.57 | **0.500** |
|  |  | -12.08 | NA | **0.000** |  |  | 4.86 | 6.02 | **0.500** |
|  |  | 12.46 | 6.47 | **0.539** |  |  | 2.83 | 7.34 | **0.500** |
| **2** | in front | 24.4 | 3.17 | **0.670** | **2** | in front | 4.65 | 7.78 | **0.500** |
|  |  | 28.64 | 3.29 | **0.613** |  |  | 4.34 | 5.62 | **0.500** |
|  |  | 15.25 | 1.09 | **0.794** |  |  | 4.1 | 9.62 | **0.500** |
| **3** | in front | -1.1 | NA | **0.000** | **3** | in front | 5.69 | 3.16 | **0.876** |
|  |  | -13 | NA | **0.000** |  |  | 7.38 | 4.59 | **0.840** |
|  |  | -8.14 | NA | **0.000** |  |  | 6.41 | 3.99 | **0.861** |
| **4** | behind | -5.83 | NA | **0.000** | **4** | behind | 7.12 | 3.87 | **0.845** |
|  |  | 39.07 | 3.89 | **0.276** |  |  | 4.45 | 3.31 | **0.903** |
|  |  | 27.27 | 4.47 | **0.495** |  |  | 8 | 5.38 | **0.826** |
|  |  |  |  |  |  |  |  |  |  |
| **Mean** |  |  |  | **0.318** |  |  |  |  | **0.679** |
| **Stdev** |  |  |  | **0.307** |  |  |  |  | **0.188** |
|  |  |  |  |  |  |  |  |  |  |
| **ID: 07** |  |  |  |  |  |  |  |  |  |
| **Pre-Training Evaluation** | | | | | **Post-Training Evaluation** | | | | |
| **Scenario** | **NearLane** | **Tgo** | **Surge** | **Safety** | **Scenario** | **NearLane** | **Tgo** | **Surge** | **Safety** |
| **1** | behind | -18.61 | NA | **0.000** | **1** | behind | 7.07 | 5.12 | **0.904** |
|  |  | -17 | NA | **0.000** |  |  | 6 | 4.09 | **0.919** |
|  |  | -23.38 | NA | **0.000** |  |  | 5.43 | 3.86 | **0.927** |
| **2** | in front | -17.38 | NA | **0.000** | **2** | in front | 4.4 | 2.88 | **0.837** |
|  |  | -14.31 | NA | **0.000** |  |  | 3.62 | 1.81 | **0.866** |
|  |  | -11.49 | NA | **0.000** |  |  | 5.07 | 3.08 | **0.812** |
| **3** | in front | -4.18 | NA | **0.000** | **3** | in front | 9.72 | 5.72 | **0.789** |
|  |  | -16.87 | NA | **0.000** |  |  | 6.23 | 6.23 | **0.865** |
|  |  | -2.25 | NA | **0.000** |  |  | 3.85 | 6.02 | **0.500** |
| **4** | behind | -5.53 | NA | **0.000** | **4** | behind | 3.74 | 7.93 | **0.500** |
|  |  | -3.98 | NA | **0.000** |  |  | 5.29 | 2.24 | **0.885** |
|  |  | -7.98 | NA | **0.000** |  |  | 1.87 | 3.85 | **0.500** |
|  |  |  |  |  |  |  |  |  |  |
| **Mean** |  |  |  | **0.000** |  |  |  |  | **0.775** |
| **Stdev** |  |  |  | **0.000** |  |  |  |  | **0.171** |
|  |  |  |  |  |  |  |  |  |  |
| **ID: 06** |  |  |  |  |  |  |  |  |  |
| **Pre-Training Evaluation** | | | | | **Post-Training Evaluation** | | | | |
| **Scenario** | **NearLane** | **Tgo** | **Surge** | **Safety** | **Scenario** | **NearLane** | **Tgo** | **Surge** | **Safety** |
| **1** | behind | 3.78 | 32.05 | **0.500** | **1** | behind | 10.12 | 4.77 | **0.863** |
|  |  | -6.87 | NA | **0.000** |  |  | -24.6 | NA | **0.000** |
|  |  | -8.81 | NA | **0.000** |  |  | 8.38 | 5.55 | **0.887** |
|  |  | -7.00 | NA | **0.000** |  |  | 5.19 | 3.12 | **0.930** |
|  |  | -16.39 | NA | **0.000** |  |  | 7.25 | 2.86 | **0.902** |
|  |  | -35.00 | NA | **0.000** |  |  | 4.34 | 2.47 | **0.941** |
|  |  | 2.7 | 28.43 | **0.500** |  |  | 7.2 | 3.03 | **0.903** |
| **2** | in front | -35.00 | NA | **0.000** | **2** | in front | 10.31 | 4.9 | **0.618** |
|  |  | -35.00 | NA | **0.000** |  |  | 9.01 | 3.1 | **0.666** |
|  |  | -0.61 | NA | **0.000** |  |  | 9.02 | 4.05 | **0.666** |
|  |  | -18.42 | NA | **0.000** |  |  | 9.93 | 3.22 | **0.632** |
|  |  | -17.78 | NA | **0.000** |  |  | 8.33 | 3.42 | **0.691** |
|  |  | 4.09 | 18.55 | **0.500** |  |  | 9.11 | 3.52 | **0.663** |
|  |  | -18.55 | NA | **0.000** |  |  | 7.42 | 2.65 | **0.725** |
| **3** | in front | -6.06 | NA | **0.000** | **3** | in front | 5.53 | 4.09 | **0.880** |
|  |  | -12.42 | NA | **0.000** |  |  | 5.76 | 2.71 | **0.875** |
|  |  | -23.08 | NA | **0.000** |  |  | 8.09 | 4.45 | **0.824** |
|  |  | -9.18 | NA | **0.000** |  |  | 5.19 | 2.81 | **0.887** |
|  |  | -7.89 | NA | **0.000** |  |  | -2.29 | NA | **0.000** |
|  |  | -6.31 | NA | **0.000** |  |  | 7.08 | 2.9 | **0.846** |
|  |  | 2.82 | 4.13 | **0.500** |  |  | 5.2 | 1.53 | **0.887** |
| **4** | behind | 1.56 | 8.06 | **0.500** | **4** | behind | 4.24 | 1.08 | **0.908** |
|  |  | -8.62 | NA | **0.000** |  |  | 3.97 | 1.43 | **0.914** |
|  |  | -19.93 | NA | **0.000** |  |  | 4.61 | 1.44 | **0.900** |
|  |  | -7.23 | NA | **0.000** |  |  | 6.23 | 1.71 | **0.865** |
|  |  | 1.81 | 7.37 | **0.500** |  |  | 3.21 | 1.6 | **0.930** |
|  |  | -18.91 | NA | **0.000** |  |  | 5.15 | 2.15 | **0.888** |
|  |  | -7.1 | NA | **0.000** |  |  | -4.83 | NA | **0.000** |
|  |  |  |  |  |  |  |  |  |  |
| **Mean** |  |  |  | **0.107** |  |  |  |  | **0.739** |
| **Stdev** |  |  |  | **0.209** |  |  |  |  | **0.280** |
|  |  |  |  |  |  |  |  |  |  |
| **ID: 11** |  |  |  |  |  |  |  |  |  |
| **Pre-Training Evaluation** | | | | | **Post-Training Evaluation** | | | | |
| **Scenario** | **NearLane** | **Tgo** | **Surge** | **Safety** | **Scenario** | **NearLane** | **Tgo** | **Surge** | **Safety** |
| **1** | behind | 4.54 | 2.88 | **0.832** | **1** | behind | 9.03 | 3.92 | **0.878** |
|  |  | 2.05 | 5.17 | **0.500** |  |  | 4.59 | 3.86 | **0.938** |
|  |  | 3.15 | 4.59 | **0.500** |  |  | 5.36 | 4.46 | **0.928** |
|  |  | 2.24 | 2.96 | **0.500** |  |  | 3.81 | 2.66 | **0.949** |
|  |  | 5.31 | 2.77 | **0.803** |  |  | 6.85 | 5.24 | **0.907** |
|  |  | 3.4 | 1.45 | **0.874** |  |  | 5.86 | 3.54 | **0.921** |
|  |  | 4.12 | 1.84 | **0.847** |  |  | 3.84 | 3.33 | **0.948** |
| **2** | in front | 5.67 | 3.9 | **0.923** | **2** | in front | 6.23 | 5.42 | **0.769** |
|  |  | 5.75 | 4.09 | **0.922** |  |  | 4.06 | 3 | **0.850** |
|  |  | -2.93 | NA | **0.000** |  |  | 4.59 | 3.77 | **0.830** |
|  |  | -1.51 | NA | **0.000** |  |  | 4.96 | 3.27 | **0.816** |
|  |  | 6.29 | 3.37 | **0.915** |  |  | 7.54 | 5.46 | **0.721** |
|  |  | 3.18 | 4.17 | **0.500** |  |  | 4.34 | 2.39 | **0.839** |
|  |  | 1.39 | 4.93 | **0.500** |  |  | 5.81 | 3.15 | **0.785** |
| **3** | in front | 19.27 | 1.44 | **0.643** | **3** | in front | 3.48 | 1.87 | **0.924** |
|  |  | -15.62 | NA | **0.000** |  |  | 3.45 | 2.09 | **0.925** |
|  |  | -13.12 | NA | **0.000** |  |  | 1.95 | 1.95 | **0.958** |
|  |  | -24.11 | NA | **0.000** |  |  | 3.95 | 2.56 | **0.914** |
|  |  | -20.03 | NA | **0.000** |  |  | 2.69 | 1.59 | **0.942** |
|  |  | -14.02 | NA | **0.000** |  |  | 3.03 | 2.21 | **0.934** |
|  |  | -13.22 | NA | **0.000** |  |  | 7.02 | 1.45 | **0.847** |
| **4** | behind | 17.71 | 1.62 | **0.672** | **4** | behind | 2.33 | 1.54 | **0.949** |
|  |  | -1.02 | NA | **0.000** |  |  | 3.07 | 1.96 | **0.933** |
|  |  | 3.91 | 2.76 | **0.928** |  |  | 3.56 | 2.54 | **0.923** |
|  |  | -12.34 | NA | **0.000** |  |  | 3.72 | 2.45 | **0.919** |
|  |  | -26.8 | NA | **0.000** |  |  | 2.31 | 2.31 | **0.950** |
|  |  | -16.5 | NA | **0.000** |  |  | 4.14 | 2.96 | **0.910** |
|  |  | -21.26 | NA | **0.000** |  |  | 4.16 | 3.35 | **0.910** |
|  |  |  |  |  |  |  |  |  |  |
| **Mean** |  |  |  | **0.388** |  |  |  |  | **0.893** |
| **Stdev** |  |  |  | **0.391** |  |  |  |  | **0.062** |
|  |  |  |  |  |  |  |  |  |  |
| **ID: 12** |  |  |  |  |  |  |  |  |  |
| **Pre-Training Evaluation** | | | | | **Post-Training Evaluation** | | | | |
| **Scenario** | **NearLane** | **Tgo** | **Surge** | **Safety** | **Scenario** | **NearLane** | **Tgo** | **Surge** | **Safety** |
| **1** | behind | 6.97 | 4.02 | **0.742** | **1** | behind | 4.99 | 3.68 | **0.933** |
|  |  | -19.52 | NA | **0.000** |  |  | 3.87 | 3.65 | **0.948** |
|  |  | -20.58 | NA | **0.000** |  |  | 8.14 | 6.79 | **0.890** |
|  |  | -17.19 | NA | **0.000** |  |  | 2.78 | 1.63 | **0.962** |
|  |  | -12.52 | NA | **0.000** |  |  | 4.67 | 2.34 | **0.937** |
|  |  | -13.81 | NA | **0.000** |  |  | 7.86 | 5.79 | **0.894** |
|  |  | -21.78 | NA | **0.000** |  |  | 4.84 | 4.25 | **0.935** |
| **2** | in front | -19.1 | NA | **0.000** | **2** | in front |  |  |  |
|  |  | -14.4 | NA | **0.000** |  |  |  |  |  |
|  |  | -19.58 | NA | **0.000** |  |  |  |  |  |
|  |  | -27.58 | NA | **0.000** |  |  |  |  |  |
|  |  | -22.06 | NA | **0.000** |  |  |  |  |  |
|  |  | -15.2 | NA | **0.000** |  |  |  |  |  |
|  |  | -14.47 | NA | **0.000** |  |  |  |  |  |
| **3** | in front | -8.37 | NA | **0.000** | **3** | in front | 2.53 | 1.8 | **0.951** |
|  |  | -1.85 | NA | **0.000** |  |  | 3.21 | 1.73 | **0.938** |
|  |  | -18.18 | NA | **0.000** |  |  | 3.27 | 2.16 | **0.937** |
|  |  | -8.37 | NA | **0.000** |  |  | 4.26 | 1.46 | **0.918** |
|  |  | -13.51 | NA | **0.000** |  |  | 2.88 | 1.69 | **0.945** |
|  |  | 5.32 | 1.9 | **0.898** |  |  | 3.48 | 1.66 | **0.933** |
|  |  | -16.1 | NA | **0.000** |  |  | 2.67 | 2.08 | **0.949** |
|  |  |  |  |  |  |  | 4.35 | 1.69 | **0.916** |
|  |  |  |  |  |  |  | 3.06 | 2 | **0.941** |
| **4** | behind | 1.97 | 4.68 | **0.500** | **4** | behind | 3.16 | 2.18 | **0.939** |
|  |  | -8.25 | NA | **0.000** |  |  | 2.16 | 1.44 | **0.958** |
|  |  | 3.46 | 2.4 | **0.933** |  |  | 3.3 | 2.4 | **0.937** |
|  |  | 12.27 | 1.68 | **0.764** |  |  | 2.94 | 1.88 | **0.943** |
|  |  | 8.62 | 1.95 | **0.834** |  |  | 2.9 | 1.71 | **0.944** |
|  |  | -23.2 | NA | **0.000** |  |  | 4.7 | 1.62 | **0.910** |
|  |  | 26.18 | 2.34 | **0.497** |  |  | 2.81 | 1.19 | **0.946** |
| **3** | behind | -1.97 | NA | **0.000** | **3** | in front | 2.83 | 2.06 | **0.938** |
|  |  | 7.04 | 3.07 | **0.870** |  |  | 5.84 | 4.31 | **0.873** |
|  |  | 1.62 | 1.19 | **0.970** |  |  | 3.79 | 3.18 | **0.918** |
|  |  | 2.22 | 2.64 | **0.500** |  |  | 4.18 | 2.84 | **0.909** |
|  |  | 1.29 | 3.15 | **0.500** |  |  | 4.34 | 3.95 | **0.906** |
|  |  | 0.87 | 1.47 | **0.500** |  |  | 4.77 | 3.65 | **0.896** |
|  |  | 2.63 | 3.73 | **0.500** |  |  | 4.37 | 3.9 | **0.905** |
| **4** | in front | 6.95 | 3.32 | **0.871** | **4** | behind | 4.59 | 3.53 | **0.900** |
|  |  | 3.21 | 2.61 | **0.941** |  |  | 4.45 | 3.9 | **0.903** |
|  |  | -6.88 | NA | **0.000** |  |  | 5.09 | 3.61 | **0.889** |
|  |  | -3.68 | NA | **0.000** |  |  | 3.9 | 3.35 | **0.915** |
|  |  | -14.71 | NA | **0.000** |  |  | 2.83 | 2.23 | **0.938** |
|  |  | -1.97 | NA | **0.000** |  |  | 3.71 | 1.64 | **0.919** |
|  |  | -1.32 | NA | **0.000** |  |  | 3.25 | 2.53 | **0.929** |
|  |  | -1.97 | NA | **0.000** |  |  | NA | NA |  |
|  |  |  |  |  |  |  |  |  |  |
| **Mean** |  |  |  | **0.252** |  |  |  |  | **0.926** |
| **Stdev** |  |  |  | **0.366** |  |  |  |  | **0.022** |
